# Epidemiology of alcohol-related emergency hospital admissions in children and adolescents: An e-cohort analysis in Wales in 2006-2011

**DOI:** 10.1371/journal.pone.0217598

**Published:** 2019-06-04

**Authors:** Laszlo Trefan, Andrea Gartner, Amy Alcock, Daniel Farewell, Jennifer Morgan, David Fone, Shantini Paranjothy

**Affiliations:** 1 Division of Population Medicine, School of Medicine, Cardiff University, Cardiff, United Kingdom; 2 Royal Gwent Hospital, Newport, United Kingdom; 3 NHS Wales Delivery Unit, Pencoed, United Kingdom; King's College London, UNITED KINGDOM

## Abstract

**Objective:**

Harmful levels of alcohol consumption in young people are prevalent and of increasing public concern in the western world. Rates of alcohol-related emergency hospital admissions in children and young people between 10 to 17 years were described, and the reasons for these admissions and their association with socio-demographic factors were examined.

**Methods:**

E-cohort data were extracted from the Secure Anonymised Information Linkage Databank, which contained alcohol-related emergency hospital admissions (N = 2968) from 2006 to 2011 in children and adolescents aged 10 to 17 years in Wales. A generalised linear mixed model was fitted using a log-link with a population offset to the data to calculate incident rate ratios (IRRSs).

**Results:**

There was a general decreasing trend from 2006 to 2011 in the number and rate of alcohol-related emergency hospital admissions; the mean age of admission was 15.4 (standard deviation 1.4) years. In each of the four youngest age groups (10–13,14,15,16 years), females had higher IRRs than males. Males had slightly higher IRR compared to females only in the oldest age group (17 years). IRRs increased with increasing deprivation. The majority (92%) of the admissions lasted one day and most of the admissions (70%) occured during the last three days of the week with a peak on Saturday. The length of stay in hospital was longer in cases when self-harm were present. Multiple admissions showed high prevalance of serious self-harm cases in females. The number of admissions with injuries and falls were higher for males than females.

**Conclusion:**

Female children and adolescents were more likely to be admitted to hospital for alcohol-related reasons. These data illustrate the significant burden of alcohol-related harm in young people and highlight the need for interventions and policies that promote safe drinking practices among young people to prevent future alcohol-related harm during the life-course.

## Introduction

Harmful levels of alcohol consumption in children and young people are prevalent in the western world and infulenced by several factors including social position among peers, family [1] and school socioeconomic status [2].

Early initiation of alcohol consumption has been shown to be linked to binge drinking [3], heavy drinking [4], alcohol drinking problems [5], alcohol use disorder [6] and alcohol dependence [7] in adult life.

Although weekly alcohol use in adolescents has decreased in the UK [8], heavy episodic underage drinking is a growing public health concern in many European [9] countries, and is associated with an increased risk of violent behaviors [10], victimization [11], injuries [12], and unwanted sexual activities [13]. According to the ESPAD (European School Survey Project on Alcohol and Other Drugs) report [14] in 2016 it is estimated that on average in Europe 35% of 15–16 year old students reported binge drinking over the past 30 days.

Alcohol consumption among children and adolescents can result in a range of negative health effects including emergency hospital attendances due to intoxication or injuries [15]. Admission rates for mental and behavioural disorders caused by alcohol in England and Wales rose slightly in women aged under 25 years and in children (aged 0–14 years) between 1998 and 2002 [16]. An understanding of the population characteristics of young people who are at highest risk of alcohol-related harm is important to inform public health interventions and policies [17]. However, little is known about the epidemiology of these admissions, associated socio-demographic factors and contributing causes for admission. There have been studies, mainly in Europe, which report country level data with up to 10 years follow up [18–22], these are mainly descriptive statistics on alcohol intoxication of adolescents attending paediatric departments using retrospective questionnaires.

In this study the epidemiology of alcohol-related harm in children who were old enough to suffer harm from intentional consumption of alcohol, in contrast to accidental ingestion is described. In line with national level data [18–23] this was defined to be at least 10 years of age. Specifically rates of alcohol-related emergency hospital admissions in children and young people between 10 and 17 years are described, along with the examination of the reasons for these admissions and how these rates vary according to socio-demographic factors.

## Methods

### E-cohort characteristics

This study used anonymised record-linked hospital admission data from the Patient Episode Database for Wales (PEDW) held in the Secure Anonymised Information Linkage (SAIL) databank at Health Data Research UK, Swansea University, UK [24]_,_[25]. PEDW records were extracted for all children, who lived in Wales on 1 January 2006, and were aged between 10 and under 18 years on admission to hospital between 1 January 2006 and 31 December 2011.

### Data source and population data

The PEDW includes demographic and clinical data on all inpatient and daycase admissions in NHS Wales hospitals and for all Welsh residents treated in England. Each patient record includes anonymised unique identifiers, the date of admission, admission method (e.g. emergency or elective), patient classification (in-patient or day case), up to 14 (ICD-10) [26] diagnosis codes (e.g. F10.0-‘Acute intoxication’, Z72.0–‘Tobacco use,’ Z72.2-‘Drug use’ in the first three positions) and discharge destination (to identify inter-hospital transfers), discharge method (to identify death in hospital), and date of discharge [27]. Each record also included the UK Census lower layer super output area (LSOA) of residence [28], which was used to link to quintiles of the Welsh Index of Multiple Deprivation 2008 (WIMD 2008) [29] and the UK Office for National Statistics (ONS) rural-urban settlement type classification [30]. Three categories were defined: the village category included ‘village, hamlet and isolated dwellings–sparse/less sparse’, town included ‘town and fringe–sparse/less sparse’ and urban included ‘urban >10k sparse/less sparse’. To ensure complete coverage of the subjects’ age, sex and LSOA code data were also used from the Welsh Demographic Service total population register, which is available through the SAIL databank, to validate the information held in PEDW.

Mid-year population estimates between 2006 and 2011 were provided by the Public Health Wales Observatory using ONS small area population estimates [31] and was offered at LSOA levels. This population data was further aggregated according to each strata of the socio-demographic variables and used in the final model.

### Information governance and research ethics

Approval for the use of anonymised data in this study was granted by an independent Information Governance Review Panel (IGRP), with membership comprised of senior representatives from the British Medical Association (BMA), the National Research Ethics Service (NRES), Public Health Wales and NHS Wales Informatics Service (NWIS).

The IGRP assesses whether research proposals for using the SAIL databank meet the strict information governance arrangements set out in the multiple data access agreements. Robust policies, structures, controls and special software are in place to protect privacy through a reliable matching, anonymisation and encryption process achieved in conjunction with NWIS, including presentation of data outside the SAIL Databank [24],[32]. The use of anonymised data for research is outside the scope of the EU General Data Protection Regulations (GDPR) and the UK Data Protection Act.

### Outcome measures

Previously defined ICD-10 codes were used to identify alcohol-related hospital admissions [33] (see S1 Table for details). In this analysis, an alcohol-related hospital admission was defined by the presence of any of these codes in the first three coding positions or in the fourth position if the first three coding positions contain only R or Z codes (excepting R78.0, Z50.2, Z71.4, Z72.1).

### Statistical analyses

Descriptive statistics were used to present the number and rates of alcohol-related emergency hospital admissions by year, age group (10–13,14,15,16,17), sex (male or female), quintile of socioeconomic disadvantage (least deprived, less-, mid-, more- and most deprived) based on WIMD 2008 categories and rural-urban settlement type (village, town, urban). The youngest age group included children aged between 10 and 13 years because this offered sufficient numbers to run the statistical models. Age was calculated at the time of the admission. Length of stay in hospital was calculated as the difference between discharge- and admission date, expressed in days. The total counts of all alcohol-related emergency admissions in each strata of the socio-demographic variables was modelled in generalised linear mixed models (GLMM) using a log-link and a population offset derived from the previously described mid-year population estimates [34]. Incident rate ratios (IRR) were estimated with 95% confidence intervals (CI). To assess how these rates vary according to age, sex and deprivation, a final model of all alcohol-related emergency admissions was fitted that included three two-way interaction terms between these variables (age*sex, age*deprivation, sex*deprivation). Loglikelihood ratios of the models [34] were used to compare model fits using likelihood ratio tests [35]. All analyses were performed using R-software version 3.2.1 [36].

Aditional analysis was carried out to produce descriptive statistics of multiple alcohol-related emergency hospital admissions.

## Results

**Fig 1:**
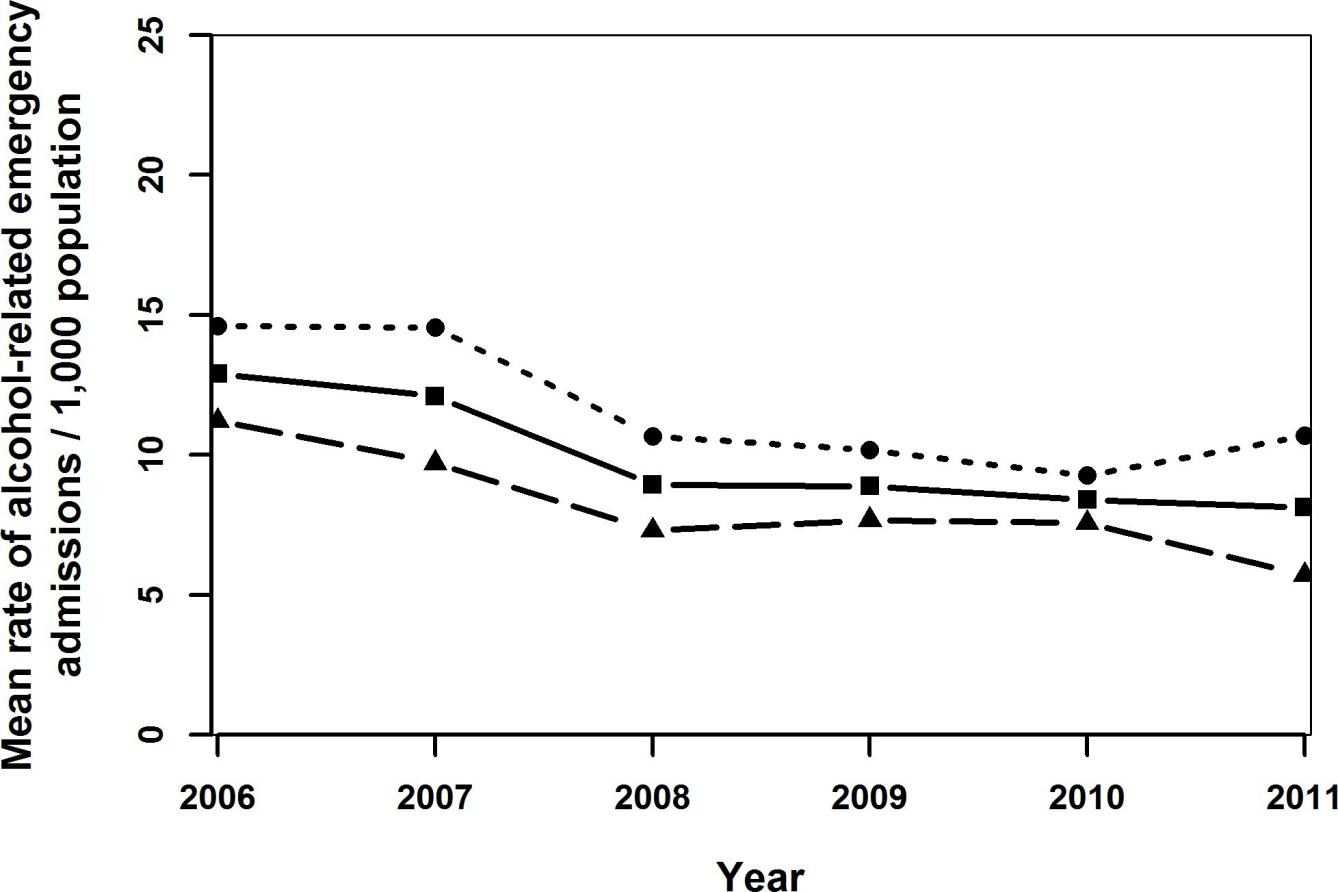
Mean rates of alcohol-related emergency hospital admissions per 1,000 population (solid line), females (dotted line) and males (dashed line) of children and adolescents aged 10–17 years in Wales in 2006–2011.

During the six-year study period, there were 2,968 alcohol-related emergency hospital admissions in 2708 children and adolescents aged between 10 and 18 years. This represents a mean annual rate of 9.9 per 1,000 population, declining over the study period, from 12.9 per 1,000 population in 2006 to 8.1 per 1,000 in 2011 (S2 Table and Fig 1). The majority of individuals (N = 2515, 93%) had only one admission during the study period, whilst the remaining 7% were admitted at least twice. Sixteen of these admissions resulted in death in hospital and six of these had an alcohol-related underlying cause of death. The mean age at admission and its standard deviation were 15.4 and 1.4 years, respectively.

**Fig 2:**
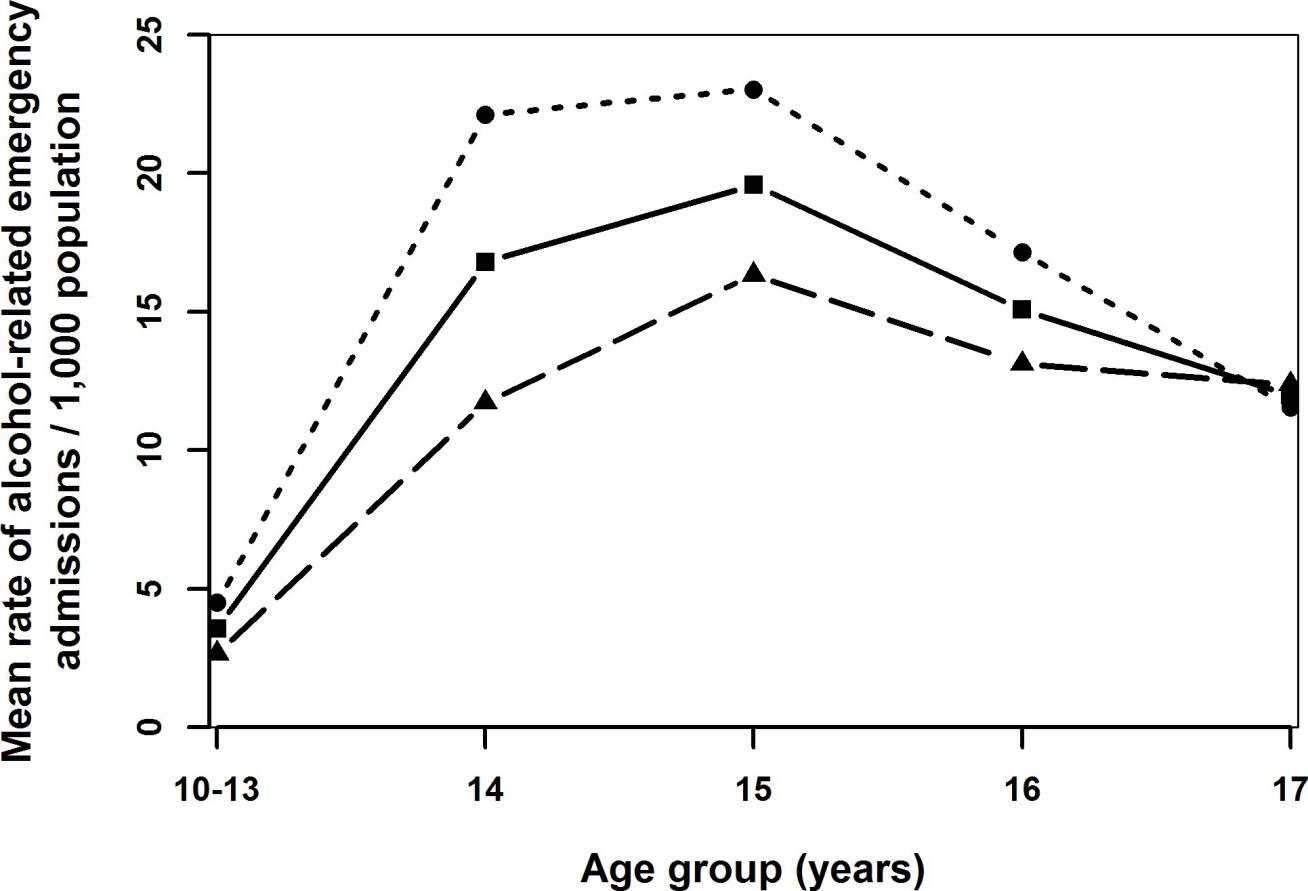
Mean rates of alcohol-related emergency hospital admissions per 1,000 population (solid line), females (dotted line) and males (dashed line) in different age groups of childen and adolescents aged 10–17 years in Wales in 2006–2011.

**Fig 3:**
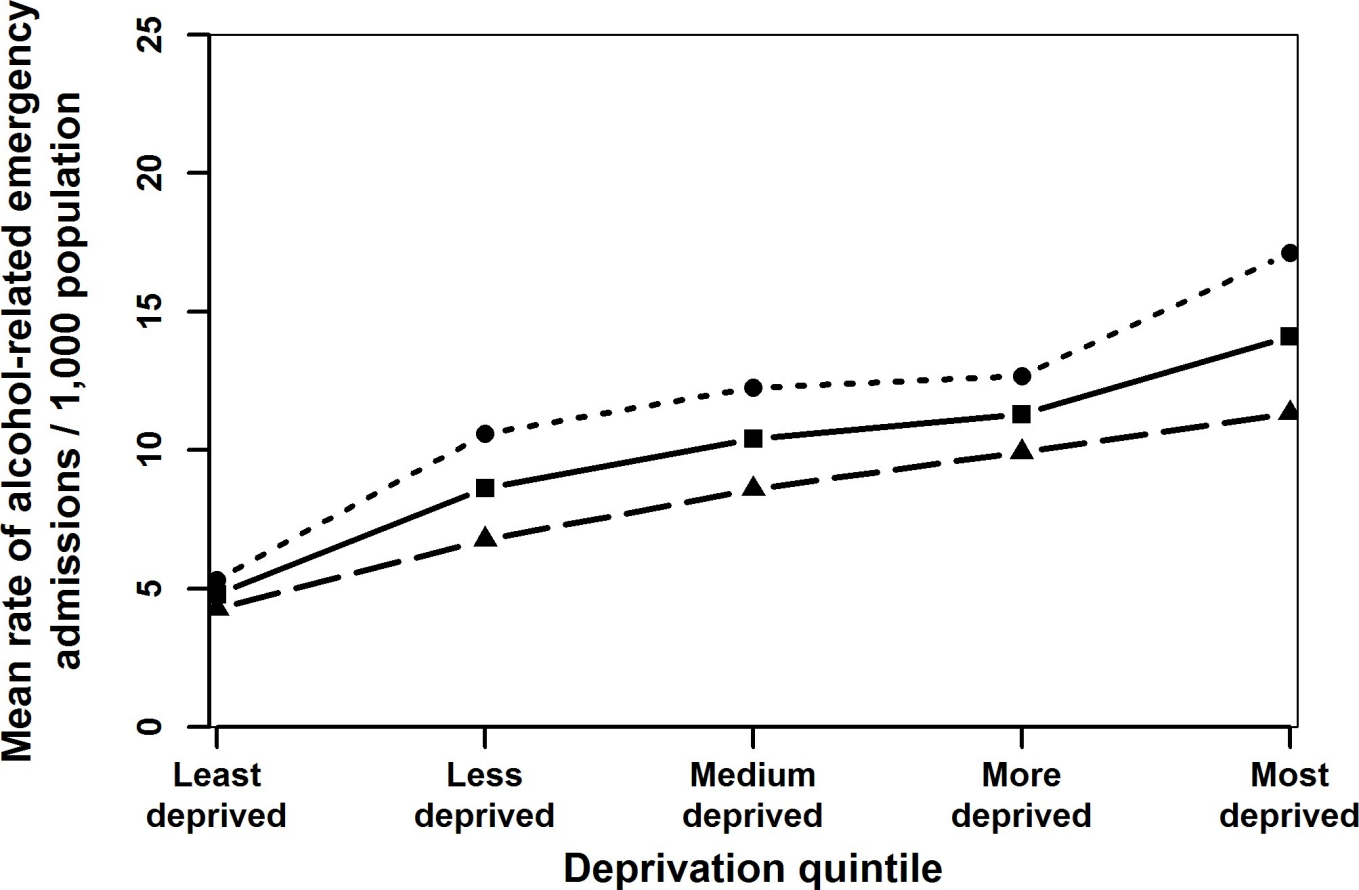
Mean rates of alcohol-related emergency hospital admissions per 1,000 population (solid line), females (dotted line) and males (dashed line) in different deprivation quintiles of children and adolescents aged 10–17 years in Wales in 2006–2011.

**Fig 4:**
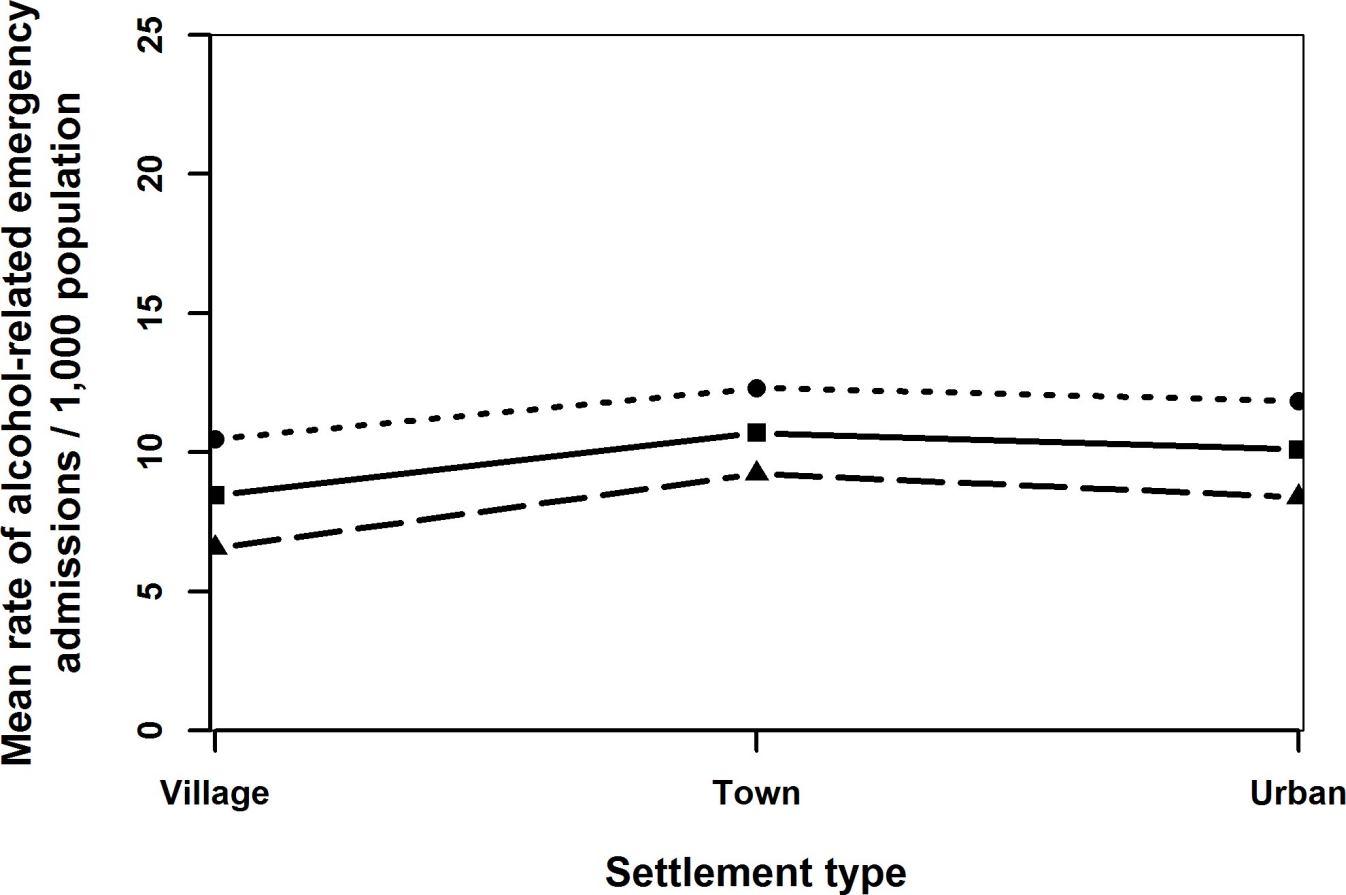
Mean rates of alcohol-related emergency hospital admissions per 1,000 population (solid line), females (dotted line) and males (dashed line) by settlement type, children and adolescensts aged 10–17 years in Wales in 2006–2011.

Generally admission rates declined over time (Fig 1) and up to age 16 years, the admission rates for females was higher than for males (Figs 1–4). Admission rates increased with increasing deprivation (Fig 3) and were higher among town compared to urban- and village dwellers (Fig 4).

**Fig 5:**
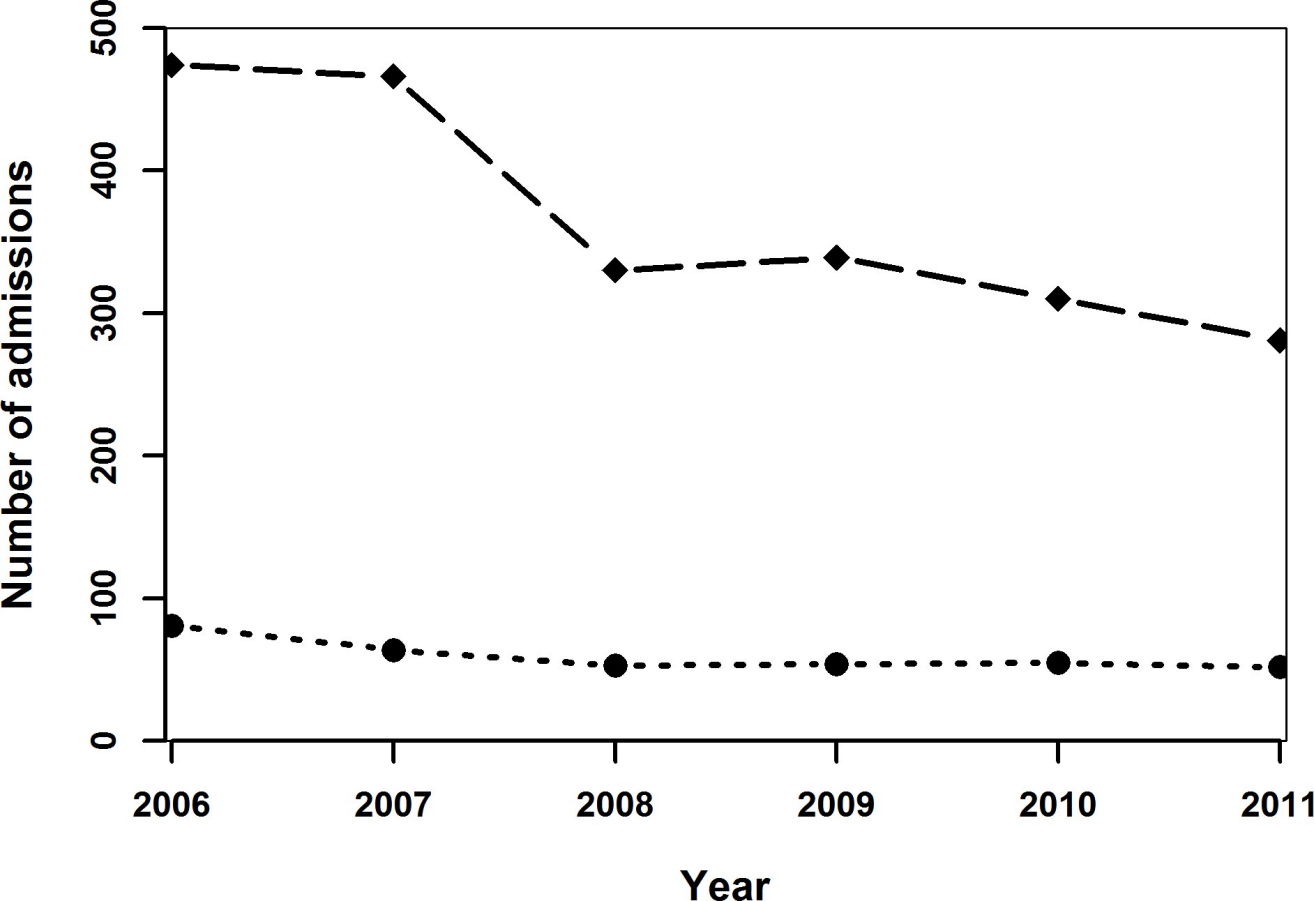
Number of the two most common types of alcohol-related emergency hospital admissions over time: Acute intoxication (dashed line) and “toxic effect: Ethanol (dotted line)”, in children and adolescents aged 10–17 years in Wales.

Almost eighty percent of alcohol-related emergency admissions in children and young people were coded as having a ‘mental and behavioural disorder related to use of alcohol’ (Table 1). Of these, the most common diagnosis of admission was acute intoxication, but there were a small number admitted with harmful use and dependence syndrome as well. Around 12% of the admissions were acute toxic effects of alcohol. There was a sharp drop in acute alcohol intoxication between 2007 and 2008, but the number of alcohol poisonings was relatively constant over time (Fig 5).

**Table 1:** Number and percentage of first alcohol code in alcohol-related emergency admissions of children and adolescents aged 10–17 years in Wales between 2006 and 2011.

Description	Number N = 2968	%
Dependence syndrome	17	0.6
Acute intoxication	2192	73.9
Harmful use	159	5.4
Toxic effect: Ethanol	359	12.1
Alcohol use	114	3.8
Accidental poising by and exposure to alcohol	47	1.6
Intentional self-poising by and exposure to alcohol	25	0.8
Alcoholic gastritis	18	0.6
Alcohol involvement, not otherwise specified	14	0.5
All others*	23	0.7

*All individual counts were <5 and so suppressed by Information Governance rules

A total of 1188 (40.0%) of the alcohol-related emergency hospital admissions had an associated coded cause(s), of which 472 (15.9%) were ‘accidents’, mainly ‘falls’ and ‘ accidental poisoning due to noxious substances’ (Table 2). There were 429 (14.5%) admissions for ‘intentional self-harm’, mainly due to ‘intentional self-poisoning by and exposure to alcohol’ and ‘intentional self-poisoning by and exposure to nonopioid analgesics, antipyretics and antirheumatics’ (Table 2). The number of ‘assults’ as contributing cuase was relatively low with only 64 admissions (Table 2).

**Table 2:** Cause(s) and their main categories associated with alcohol-related emergency hospital admissions of children and adolescents aged 10–17 years in Wales, 2006–2011.

**External cause of morbidity and mortality**	**Number of** **admissions**
***Accidents***	***472***
Falls	184
Accidental poisoning to noxious substances	174
***Intentional self-harm***	***429***
Intentional self-poisoning by and exposure to alcohol	273
Intentional self-poisoning by and exposure to nonopioid analgesics, antipyretics and antirheumatics	152
***Assults***	***64***
**Injury, poisoning and certain other consequences of external causes**	
***Injuries***	***426***
Injuries to the head	316
Injuries to the wrist and hand	51
Injuries to the knee and lower leg	30
***Poisoning by drugs*, *medicaments and biological substances***	***422***
Poisoning by non-opiod analgesics, antipyretics and antirheumatics	184
Poisoning by psychotropic- drugs, not elsewhere classified	73
Poisoning by antiepileptic, sedative-hypnotic and antiparkinsonism drugs	61
***Toxic effects of substances chiefly nonmedicinal as to source***	***436***
Toxic effect of alcohol: Ethanol	359
Toxic effect of alcohol: unspecified	66

In 426 (14.4%) of the alcohol-related emergency hospital admissions there was a record of associated injuries coded. The majority of these were head injuries while the rest were injuries to limbs (Table 2). 422 (14.2%) of the admissions were associated with ‘poisoning by drugs, medicaments and biological substances’ of which the three most common were ‘non-opiod analgesics, antipyretics and antirheumatics’, ‘psychotropic- drugs, not elsewhere classified’ and ‘antiepileptic, sedative-hypnotic and antiparkinsonism drugs’ (Table 2). There were 436 (14.7%) admissions, which were associated with ‘toxic effects of substances chiefly nonmedicinal as to source’, most of them were due to alcohol (Table 2).

Almost all ‘intentional self-poisoning by and exposure to alcohol’ cases were due to ‘toxic effect of alcohol’ and almost all cases of ‘intentional self-poisoning by and exposure to nonopioid analgesics’ were due to poisoning by ‘non-opiod analgesics, antipyretics and antirheumatics’ (Table 2).

Although overall there were more admissions for females than for males, this was not the case for admissions with a contributory cause of injuries, head injuries or falls. The number of females vs. males in these categories were 217 vs. 245, 124 vs. 192 and 88 vs. 96, respectively.

The incident rate ratios of alcohol-related emergency hospital admissions in children and adolescents decreased during the six year study period (Table 3), but varied according to age and sex (Table 4). The model showed higher incident rate ratios for young females of age groups 14,15 and 16 years compared to incident rate ratios of young males for the same age groups. For one age group (17 years) the incident rate ratio was slightly higher for males (Table 4). Generally the incident rate ratio was higher for young females than young males and increased by deprivation. Little effect of residential settlement type was found.

**Table 3:** Incident rate ratios of emergency admissions from alcohol-related conditions (adjusted by sex and age interaction) by year, children and adolescents aged 10–17 years in Wales, 2006–2011.

	Incident rate ratio (95% confidence interval)	p-value
**Year**		
**2006**	Reference	
**2007**	0.95 (0.84,1.08)	0.27
**2008**	0.69 (0.61,0.79)	<0.001
**2009**	0.7 (0.61,0.79)	<0.001
**2010**	0.66 (0.57,0.75)	<0.001
**2011**	0.62 (0.53,0.72)	<0.001
**Deprivation quintile**		
**Least deprived**	Reference	
**Less deprived**	1.96 (1.65,2.32)	<0.001
**Middle deprived**	2.28 (1.94,2.67)	<0.001
**More deprived**	2.41 (2.07,2.81)	<0.001
**Most deprived**	2.83 (2.42,3.3)	<0.001
**Settlement type**		
**Village**	Reference	
**Town**	1.15 (0.99,1.33)	<0.05
**Urban**	1.01 (0.9,1.14)	0.33

**Table 4:** Incident rate ratios of emergency admissions from alcohol-related conditions of children and adolescents aged 10–17 years in Wales, 2006–2011.

	Female	Male	p-value
Age group	Incident rate ratio (95% confidence interval)	Incident rate ratio (95% confidence interval)	
**10–13**	1.55 (1.27,1.9)	Reference	
**14**	7.92 (6.35,9.87)	4.26 (3.43,5.29)	<0.001
**15**	7.64 (6.12,9.53)	5.64 (4.61,6.9)	<0.001
**16**	4.81 (3.78,6.12)	4.28 (3.43,5.33)	<0.001
**17**	2.46 (1.85,3.28)	3.77 (3.01,4.72)	<0.001

The analysis of length of stay in hospital showed that almost 60% of the alcohol-related emergency admissions were shorter than a day and 92% of them were less than 2 days (Table 5). It was also found that this pattern is true for the previously described associated injuries and cause(s) except in ‘intentional self-poisoning by and exposure to nonopioid analgesics, antipyretics and antirheumatics’ cases, where 38.8%, 42.1%, 9.2%, 9.9% of the 152 admissions related to 0, 1, 2, 3 days or longer in hospital, respectively.

**Table 5:** Length of stay in hospital (in days) of children and adolescents aged 10–17 years with alcohol-related emergency hospital admissions in Wales, 2006–2011.

Length of stay in hospital	Number of admission	Percentage of total admission
**0 day**	1719	57.9%
**1 day**	1012	34.1%
**2days**	145	4.9%
**3 days or longer**	92	3.1%
**Total**	2968	

**Fig 6:**
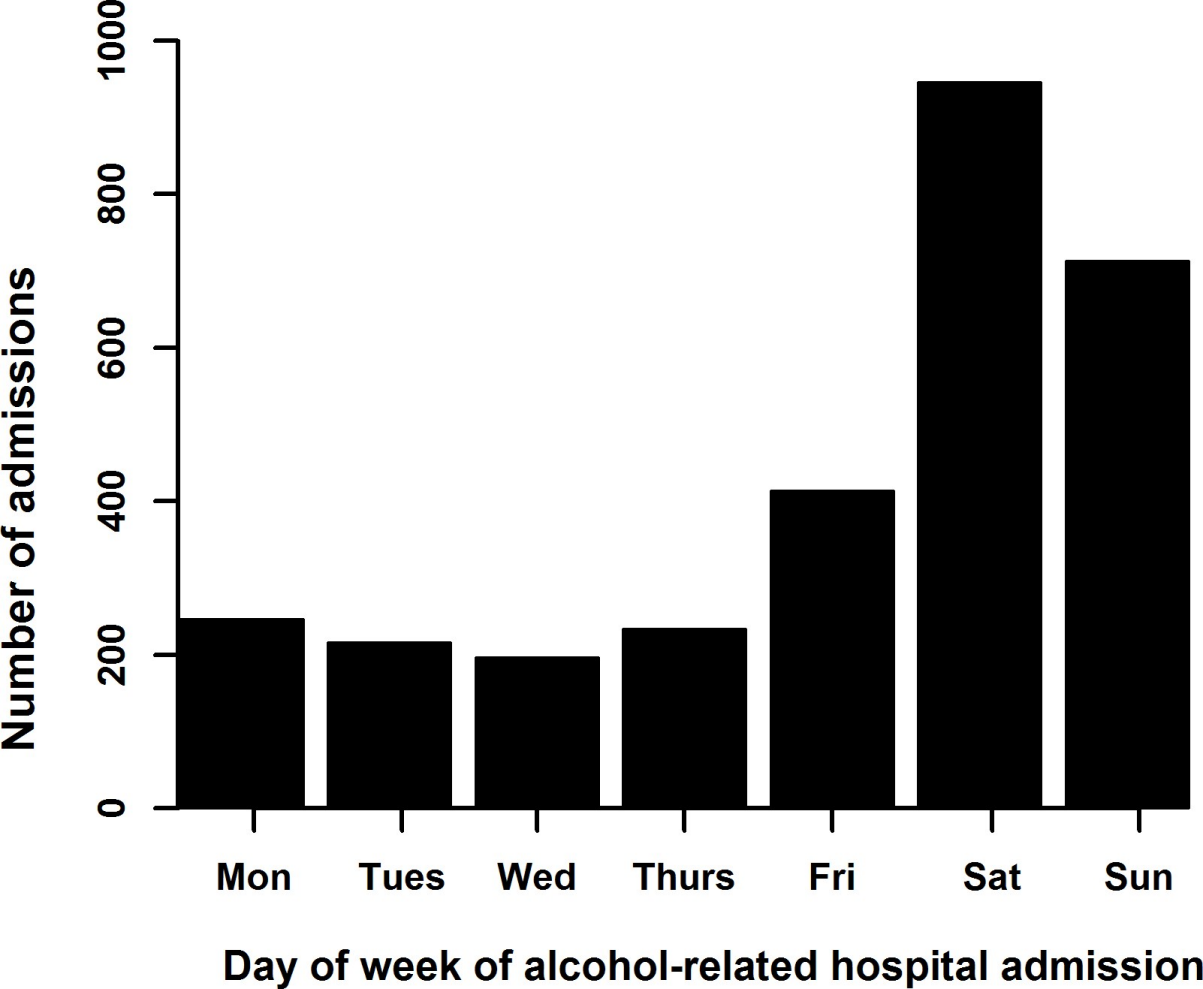
The day of the week of alcohol-related emergency hospital admissions of children and adolescents aged 10–17 years in Wales, 2006–2011.

Further time analysis showed that almost 70% of the admissions happened in the last three days (Friday/Saturday/Sunday) of the week and there was a peak of admissions on Saturdays (Fig 6).

During the six-year period, there were 193 children and adolescents who had 453 multiple alcohol-related emergency hospital admissions, of which 82.9%, 10.9%, 2.6% and 3.6% of the persons had 2, 3, 4 and at least 5 admissions, respectively. The mean age at admission and its standard deviation were 15.6 and 1.3 years, respectively. Generally these admissions showed the same trends as the previously described all admissions. However, there were a few exceptions. There was slighly higher number of annual multiple admissions in 2010 (n = 72) than either in 2008 (n = 61) or 2009 (n = 58) or 2011 (n = 63), probably due to higher numbers of acute intoxications in 2010 (n = 55) than in 2008 (n = 43), 2009 (n = 39) and 2011 (n = 37). The length of stay in hospital also showed a slight shift to longer stay in hospital, 87% of these multiple admissions were less than 2 days, 6.6% and 6.4% were 2 days, 3 days or longer, respectively. This shift was also detected in the cases when these admisions were associated with ‘intentional self-poisoning by and exposure to nonopioid analgesics, antipyretics and antirheumatics’ where 38.7%, 35.5%, <10%, <20% (numbers are suppressed by Information Governance rules) of these 31 admissions related to 0, 1, 2, 3 days or longer in hospital, respectively. When the ratio of the number of admissions of females and males were calculated in these multiple admissions, it was almost double (2.4) comparing it to the same ratio of all admissions (1.3). These female-male ratios were especially high when these admissions were associated with intentional self-harm cases, namely ‘intentional self-poisoning by and exposure to nonopioid analgesics, antipyretics and antirheumatics’ (<15.0) and 'intentional self-poisoning by and exposure to other and unspecified drugs, medicaments and biological substances' (<10.0) (numbers are suppressed by Information Governance rules)—these ratios were more than three times higher than relevant ratios, 4.6 and 3.0 of all admissions. Therefore there is an indiciation in these results of rather more serious cases of alcohol-related harm in females.

## Discussion

Our analysis showed a decreasing trend in alcohol-related emergency hospital admissions in children and young people in Wales. However, a substantial number of children and young people continue to be admitted to hospital for alcohol related harms. Children who lived in areas with higher levels of social deprivation and girls aged 10–16 years old were at higher risk of these types of hospital admissions. The vast majority of the admissions were shorter than two days in hospital and most of the admissions happened during the last three days of the week with a peak on Saturdays. The analysis of the multiple alcohol-related hospital admissions showed an association with more serious, intentional self-harm cases, that was more prevalent among females. These data provide some insights into at-risk groups that could be better targetted with public health interventions.

Our findings are mainly consistent with other studies in Europe. The mean age at admission and its standard deviation were found 15.4- and 1.4 years, respectively in our analysis, which is consistent with the findings in paediatric departments of Dutch hospitals, reporting a mean age between 14.9–15.5 years with standard deviation between 1.2–2.0 years [18–22]. A significant increase of mean age at admission was found in 4 years- [15] and 10 years long follow up [22] of these studies, which was not found by this work.

The latest follow up study on alcohol intoxication of young people in the Netherlands reported on average 0.90 days hospital stay with standard deviation of 0.41 days over 10 years [22], in our study these were 0.74 and 5.03, respectively. The differences especially in standard deviations are probably due to the differences in reason for admissions in our study, where a significant number of admissions had serious causality cases that required longer hospital stay.

The analysis of length of stay in hospital also showed that the 92% of the admissions were less than 2 days, van Hoof et al. (2010) [18] reported 85% for the same measure for Dutch hospitalised patients in 2007. The slight difference could be due to a number of reasons including non-universal coverage of Dutch hospitals data [18], differences in healthcare systems, clinical management and calculation difference. It was found that admissions with intentional self-poisoning by or exposure to nonopioid analgesics, antipyretics and antirheumatics had a longer length of hospital stay, reflecting the clinical investigations (e.g. blood tests) and management (e.g. psychiatric assessment) necessary for these types of admissions. Our analysis also showed that the majority of the admissions happened on the last three days of the week, with a peak of admissions on Saturdays, which is a similar pattern to adult alcohol-related emergency admissions [37]. For adolescents, who were admitted to hospital with alcohol intoxication in Dutch paediatric departments, Van Hoof et al. (2010) [18] and Van Zanten et al. (2013) [20] reported 23- and 15-fold glasses per day of drinks for weekend days (Friday-Sunday) comparing to weekdays. Indirectly these are in agreement with our findings and provide some explanation for them.

Although the rate of the alcohol-related emergency admission has declined over the six year study period there are still high numbers of people being admitted. The UK is consistently classed as a high prevalence country for under-age alcohol use as the proportions of pupils reporting lifetime alcohol use (90% vs 87% ESPAD (2011) [38] average for 36 European countries), use in the last 12-months (85% vs 79% ESPAD (2011) [38] average for 36 European countries) and use in the last 30-days are all higher than the ESPAD average (65% vs 57% ESPAD (2011) average for 36 European countries) [39]. ESPAD reports are based on the Monitoring Future Survey (MTF), which is an annual survey among 8^th^,10^th^ and 12^th^ grades in the United States, so their data are comparable between countries [40]. In 2007 a comparison of the MTF and ESPAD surveys showed much higher intoxication rates for the UK compared to the USA (33% vs. 18%, respectively—intoxication in the last 30-days) and 24% reported intoxication before the age of 13-years compared to 8% for the USA [39]. British youth were found to have the most positive expectations of drinking and distinctly favourable attitudes towards intoxication compared with youth in other countries in Europe and elsewhere [38, 39, 41, 42]. These findings may offer some explanations for our results.

This study found that females generally and females in three age groups (14-,15-,16- years) had higher incident rate ratios of alcohol-related emergency hospital admissions than males and youngest males (10–13 years). This incident rate ratio is only slightly higher for the oldest age group (17 years) for males than females.

Williams et al. (2005) [16] reported increasing hospital admission rates (per 100,000 population) of primary diagnosis of ‘mental and behavioural disorders due to use of alcohol’ for females with age 0–14 years in England and Wales between 2000 and 2002. A report by Public Health Wales [43] using a very similar alcohol-specific admission definition to ours found that 3-year crude rate (per 100,000) for alcohol-specific admissions of those aged under 18 decreased from 2005/06-2007/08 to 2010/11-2013/13 and were higher in each (rolling 3 years) for females than males in Wales. This pattern is similar to our findings. The latest report by Public Health England for under 18 years olds [44] found that the number of alcohol-specific admissions decreased from 2006/07 to 2014/15 and each year these numbers were higher for females than males. The report shows exactly the same tendencies for age of 10-, 11–12-,13-,14-,15-,16-,17- years between 2006/07 and 2014/15 as this study (comparison not shown) where females have a higher number of admission except 17 years olds. The report also showed a decrease of admissions for intoxication and a relatively stable number of ethanol poisonings as in our study. The herein discussed results are also in agreement with the findings of trends in alcohol-related hospital admissions in England between 2002/03 and 2013/14 [23]. In that work for acute, wholly attributable to alcohol admissions, which was a stricter defintion of alcohol-related admission than ours, declining admission rates (per 100,000) were found for age groups 10-14- and 15–19 years for both sexes over the whole period and for females aged 15–19 years the highest admission rates were found in the middle of the study period (from 2004/05 to 2010/11). In the Netherlands between 2007 and 2016 the number of young people (aged under 18 years), who were admitted hospital with alcohol intoxication increased annually from 2007 to 2011, after which it became reasonably stable, with a peak in 2015 [22]. In the same country except in 2009 and 2016 slightly higher frequencies were reported for males than females for hospital admissions of adolescents (aged under 18 years) due to alcohol intoxication or alcohol unconsiousness in paediatric hospital departments [18–22]. However, over a 10 year period in the Netherlands there were significantly more female cases with hospital alcohol intoxication treatments in age group 13 and 14 years than male cases while in age group 16 and 17 years this was the opposite [22]. The higher numbers of males in the older age groups were explained by the more experimental nature of teenage boys than girls at those ages in the Netherlands [21]. The herein discussed differences might be due to country specific reasons and it is important to note that these latter works published results of retrospective questionnaires sent to different (general, academic) types of paediatric departments in the Netherlands and response rates varied between 79.9% and 93.0%. Rapid evidence synthesis [39], which summarised the trends of underage drinking in the UK, found that there is a tendency in ESPAD reports that girls reported more drunkeness and binge drinking than boys in the UK, Norway, Denmark and Iceland. In other European countries this tendency was not apparent.The same work also found based on a government report about alcohol harm reduction strategies with children and young people that “girls are also 1.3 times more likely than boys to be admitted to hospital via the emergency department for an alcohol specific condition” [39], which is in agreement with the findings of this work. In a wider context liberalisation of gender roles has been associated with an increase in substance use and harm, including alcohol [45].

A clear gradient of increasing incident rate ratio associated with living in deprived areas of Wales was found. The incident rate ratio of admission was over three-times higher in the most deprived compared to the least deprived areas of Wales and higher in ‘town’ settlement type areas. The works which reported country-level alcohol-related hospital admissions apart from sex studied other socioeconomic aspects (education, siblings) [18–20] than this study. Healey et al. (2014) [39] stated that binge drinking patterns are more likely amongst girls and drinkers from more deprived areas of the UK, which might be reflected in our results. There is evidence that adults in deprived areas have greater alcohol-related harm for comparable levels of alcohol consumption and other factors, usually termed the alcohol harm paradox [46]. It is possible that this is reflected in our findings on increased rate ratios in deprived children and adolescents. The above mentioned Public Health England report found some evidence for the alcohol harm paradox for adolescents of aged 15 years related to admissions for alcohol specific conditions in England [44]. However, future research linking consumption in adolescents and alcohol-related harm would be needed to investigate this further.

Considering settlement type, more alcohol intoxication cases of children and adolescents (under age of 18 years) were found among urban- than rural residents over a 10 years period [47], which agrees with our findings. Although the comparison is very limited by the fact that study reported cases at a level of a paediatric department of a Polish university hospital. There is an indication in the data that most of the emergency alcohol-related admissions were due to acute intoxication or toxic effect of ethanol, (these made up at least 86% of the admissions). 14.5% of the admissions were associated with intentional-self harm. The previously mentioned report by Public Health England [44] also stated that those young people who do seek treatment for their substance misuse have a range of related problems and vulnerabilities that are likely to have an impact on their substance use, including self-harming, which is in agreement with our findings. In other analyses of survey data collected by ‘Communities that Care’ from 16 urban schools in Wales in 11 to 16 year olds, it was found that the strongest predictor of alcohol consumption was ‘family functioning’, measured by four sub-scales of which ‘parental monitoring’ and ‘family closeness’ were associated with lower consumption and ‘family conflict’ and ‘family violence’ were associated with higher consumption [48]. By identifying special group(s), the herein discussed results might help targeting interventions for underage alcohol use. The following intervention types could be considered for this purpose a.) selective intervention, which targets individuals or population subgroups that have a higher than average risk of a problem due to certain biological, psychological or social risk factors; b.) indicated intervention, which targets those already using or engaged in other highrisk behaviours to prevent more severe problems such as those attending emergency departments for alcohol-related harm or those who participate in high risk drinking behaviours; c.) identification and brief advice (IBA) in emergency departments, which is a brief intervention aiming at children and adolescents who are at risk through drinking above the recommended guidelines [49, 50].

Loxley et al. (2005) [51] suggested a model for risky substance use and related harms for adolescents in a harm-reduction framework approach [40]. Apart from neurobiological damage, social-, developmental problems, availability of substances, levels of problems with crime, antisocial behaviour and mental health have an effect on levels of harm from substance abuse. However, this work was able to consider some social aspects there was some indication in the data for mental health problems among those who attended hospital emergency departments.

The main strength of our study is the total population-based approach over a six-year time period. This resulted in a large enough number of the emergency admission outcomes to facilitate a fully stratified descriptive analysis and a robust modelling approach with sufficient precision. The PEDW dataset undergoes rigorous quality checking and has been used in previous epidemiological studies [52],[53]. Although all records had full information on age, gender and LSOA code there are known limitations with the coding of hospital data, in particular the quality of clinical coding of diagnoses. This includes records with missing diagnoses, and variation of coding amongst care providers. Peng et al. (2018) [54] found overall agreement (82.2%) and reliability (0.82) among 11 hospitals in emergency department ICD-10 (4-digits level) codes. Also, in this work the first 4 out of possible 14 (ICD-10) diagnoses codes in the PEDW dataset were used because in a previous study it was found the most optimal set of codes for similar analyses of alcohol-related emergency admissions for adults [33].

Individuals who were admitted more than once during the study period were included in the analysis to facilitate comparison of mean admission rates. The impact of this was assessed by repeating the analysis with each individual subject included only once over the time period and found no change in the results. Separately, multiple admissions were analysed, in this case desctiptive statistics were given because there were not sufficient numbers for each strata to run a model. The PEDW dataset does not include information on individual socioeconomic position so it was not posssible to investigate the relationship between this, the quintile of deprivation assigned by residence, and the study outcomes.

## Conclusions

This work studied alcohol-related emergency hospital admissions of children and adolescents aged 10 to 17 years in Wales between 2006 and 2011. Although there was a decreasing trend in the rate of these admissions, the absolute number of admissons is still high in Wales. The results show that generally females had higher incident rate ratios up to the age of 16 years. Higher deprivation and living in towns are further factors for higher incident rate ratios. The results also show that in a significant number of cases intentional self-harm were present. The vast majority of the length of admissions were within a day, however, at the present of self-harm this length was slightly longer. The number of alcohol-related admissions with injuries, head injuries and falls recorded were higher for males than females. These findings show that alcohol-related harm is still prevalent among children and young people and better targeted intervention practices and policies are needed.

## Supporting information

S1 Table ICD-10 Codes, which define alcohol-related hospital admission adopted from Fone et al (2016). (DOCX)

S2 Table Number and mean rate of emergency alcohol-related hospital admissions in children and adolescent aged 10–17 years in Wales between 2006–2011 by year and socio-demographic characteristics. (DOCX)

## Abbreviations

ESPAD: European School Survey Project on Alcohol and Other Drugs
PEDW: Patient Episode Database for Wales
SAIL: Secure Anonymised Information Linkage
UK: United Kingdom
ICD-10: International Classification of Disease, revision 10
WIMD: Welsh Index of Multiple Deprivation
ONS: UK Office for National Statistics
LSOA: UK Census lower layer super output area
IGRP: Information Governance Review Panel
BMA: British Medical Association
NRES: National Research Ethics Service
NHS: National Health Service
NWIS: Public Health Wales and NHS Wales Informatics Service
EU: European Union
GDPR: EU General Data Protection Regulations
GLMM: generalised linear mixed model
IRR: incident rate ratio
CI: confidence interval
MTF: Monitoring Future Survey
IBA: identification and brief advice
