# Supplementary material for: Epidemiology of alcohol-related emergency hospital admissions in children and adolescents: An e-cohort analysis in Wales in 2006-2011
Source: PLoS One. 2019 Jun 4;14(6):e0217598. doi: 10.1371/journal.pone.0217598 (PMC6548373; doi:10.1371/journal.pone.0217598)
Supplement: S1 Table — (DOCX) [file pone.0217598.s001.docx]

**S1 Table: ICD-10 Codes, which define alcohol-related hospital admission adopted from Fone et al (2016)***

| **ICD-10code** | **Description** |
| --- | --- |
| E24.4 | Alcohol-induced pseudo-Cushing's syndrome |
| E51.2 | Wernicke's encephalopathy |
| F10.0 | Acute intoxication |
| F10.1 | Harmful use |
| F10.2 | Dependence syndrome |
| F10.3 | Withdrawal state |
| F10.4 | Withdrawal state with delirium |
| F10.5 | Psychotic disorder |
| F10.6 | Amnesic syndrome |
| F10.7 | Residual and late-onset psychotic disorder |
| F10.8 | Other mental and behavioural disorders |
| F10.9 | Unspecified mental and behavioural disorder |
| G31.2 | Degeneration of nervous system due to alcohol |
| G40.5 | Special epileptic syndromes – if paired with other alcohol code |
| G62.1 | Alcoholic polyneuropathy |
| G72.1 | Alcoholic myopathy |
| I42.6 | Alcoholic cardiomyopathy |
| K29.2 | Alcoholic gastritis |
| K70.0 | Alcoholic fatty liver |
| K70.1 | Alcoholic hepatitis |
| K70.2 | Alcoholic fibrosis and sclerosis of liver |
| K70.3 | Alcoholic cirrhosis of liver |
| K70.4 | Alcoholic hepatic failure |
| K70.9 | Alcoholic liver disease, unspecified |
| K85.2 | Alcohol-induced acute pancreatitis |
| K86.0 | Alcohol-induced chronic pancreatitis |
| O35.4 | Maternal care for (suspected) damage to fetus from alcohol |
| R78.0 | Finding of alcohol in blood |
| T51.0 | Toxic effect: Ethanol  Excl.: acute alcohol intoxication or "hangover" effects (F10.0), drunkenness (F10.0), pathological alcohol intoxication (F10.0) |
| X45.0 | Occurrence at home |
| X45.1 | Occurrence in residential institution |
| X45.2 | Occurrence at school other institution / public admin area |
| X45.4 | Occurrence on street / highway |
| X45.5 | Occurrence at trade / service area |
| X45.6 | Occurrence at industrial / construction area |
| X45.8 | Occurrence at other specified place |
| X45.9 | Occurrence at unspecified place |
| X65.0 | Occurrence at home |
| X65.1 | Occurrence in residential institution |
| X65.2 | Occurrence at school other institution / public admin area |
| X65.4 | Occurrence on street / highway |
| X65.5 | Occurrence at trade / service area |
| X65.6 | Occurrence at industrial / construction area |
| X65.8 | Occurrence at other specified place |
| X65.9 | Occurrence at unspecified place |
| Y15.0 | Occurrence at home |
| Y15.2 | Occurrence at school other institution / public admin area |
| Y15.4 | Occurrence on street / highway |
| Y15.8 | Occurrence at other specified place |
| Y15.9 | Occurrence at unspecified place |
| Y90.0 | Blood alcohol level of less than 20 mg/100 ml |
| Y90.1 | Blood alcohol level of 20-39 mg/100 ml |
| Y90.2 | Blood alcohol level of 40-59 mg/100 ml |
| Y90.3 | Blood alcohol level of 60-79 mg/100 ml |
| Y90.4 | Blood alcohol level of 80-99 mg/100 ml |
| Y90.5 | Blood alcohol level of 100-119 mg/100 ml |
| Y90.6 | Blood alcohol level of 120-199 mg/100 ml |
| Y90.7 | Blood alcohol level of 200-239 mg/100 ml |
| Y90.8 | Blood alcohol level of 240 mg/100 ml or more |
| Y90.9 | Presence of alcohol in blood, level not specified |
| Y91.0 | Mild alcohol intoxication |
| Y91.1 | Moderate alcohol intoxication |
| Y91.2 | Severe alcohol intoxication |
| Y91.3 | Very severe alcohol intoxication |
| Y91.9 | Alcohol involvement, not otherwise specified |
| Z50.2 | Alcohol rehabilitation |
| Z71.4 | Alcohol abuse counselling and surveillance |
| Z72.1 | Alcohol use |

*Fone D, Morgan J, Fry R, et al. Change in alcohol outlet density and alcohol-related harm to population health (CHALICE): a comprehensive record-linked database study in Wales. Public Health Res 2016; 4(3): 1-222.
