# Supplementary material for: Epidemiology of alcohol-related emergency hospital admissions in children and adolescents: An e-cohort analysis in Wales in 2006-2011
Source: PLoS One. 2019 Jun 4;14(6):e0217598. doi: 10.1371/journal.pone.0217598 (PMC6548373; doi:10.1371/journal.pone.0217598)
Supplement: S2 Table — (DOCX) [file pone.0217598.s002.docx]

**S2 Table: Number and mean rate of emergency alcohol-related hospital admissions in children and adolescent aged 10-17 years in Wales between 2006-2011 by year and socio-demographic characteristics**

|  | **Number of admissions**  **(Female)** | **Mean rate/**  **1,000**  **(Female)** | **Number of admissions**  **(Male)** | **Mean rate/**  **1,000**  **(Male)** | **Number of admissions** | **Mean rate/**  **1,000** |
| --- | --- | --- | --- | --- | --- | --- |
|  |  |  |  |  |  |  |
| **Year** |  |  |  |  |  |  |
| **2006** | 365 | 14.61 | 297 | 11.21 | 662 | 12.9 |
| **2007** | 361 | 14.55 | 255 | 9.71 | 616 | 12.1 |
| **2008** | 262 | 10.67 | 189 | 7.3 | 451 | 8.94 |
| **2009** | 245 | 10.17 | 195 | 7.68 | 440 | 8.89 |
| **2010** | 218 | 9.27 | 188 | 7.57 | 406 | 8.4 |
| **2011** | 251 | 10.68 | 142 | 5.71 | 393 | 8.13 |
|  |  |  |  |  |  |  |
| **Age group** |  |  |  |  |  |  |
| **10-13** | 316 | 4.51 | 197 | 2.67 | 513 | 3.57 |
| **14** | 408 | 22.11 | 228 | 11.73 | 636 | 16.79 |
| **15** | 430 | 23.02 | 323 | 16.33 | 753 | 19.58 |
| **16** | 325 | 17.14 | 264 | 13.12 | 589 | 15.07 |
| **17** | 223 | 11.55 | 254 | 12.38 | 477 | 11.98 |
|  |  |  |  |  |  |  |
| **Sex** |  |  |  |  |  |  |
| **Male** |  |  |  |  | 1266 | 8.24 |
| **Female** |  |  |  |  | 1702 | 11.7 |
|  |  |  |  |  |  |  |
| **Deprivation quintile** |  |  |  |  |  |  |
| **Least** | 149 | 5.32 | 128 | 4.28 | 277 | 4.79 |
| **Less** | 303 | 10.59 | 204 | 6.77 | 507 | 8.63 |
| **Mid** | 356 | 12.25 | 265 | 8.59 | 621 | 10.4 |
| **More** | 369 | 12.68 | 304 | 9.93 | 673 | 11.3 |
| **Most** | 525 | 17.11 | 365 | 11.33 | 890 | 14.1 |
|  |  |  |  |  |  |  |
| **Settlement type** |  |  |  |  |  |  |
| **Village** | 257 | 10.47 | 171 | 6.57 | 428 | 8.46 |
| **Town** | 323 | 12.31 | 257 | 9.23 | 580 | 10.7 |
| **Urban** | 1122 | 11.85 | 838 | 8.39 | 1960 | 10.1 |
